# Canal complexity of a mandibular first molar

**DOI:** 10.4103/0972-0707.53341

**Published:** 2009

**Authors:** S Poorni, RA Kumar, R Indira

**Affiliations:** Department of Conservative Dentistry and Endodontics, Ragas Dental College, Chennai, India

**Keywords:** Canal complexity, mandibular first molar, middle mesial canal

## Abstract

The endodontic treatment of a mandibular molar with aberrant canal configuration can be diagnostically and technically challenging. This case report presents the treatment of a mandibular first molar with five root canals, of which three were located in the mesial root. A third canal was found between the mesiobuccal and mesiolingual root canals. The morphological pattern of separate apical terminations of three mesial root canals with separate orifices, as manifested in this case, is a rare one.

## INTRODUCTION

Knowledge of internal dental morphology is an extremely important step in planning and administering endodontic therapy. The numerous anatomical variations existing in the root canal system may contribute to the failure of root canal therapy.[1] Knowledge of the most common anatomical characteristics and their possible variations is fundamental because nontreatment of even one canal can lead to endodontic treatment failure.[2]

The mandibular first molar which is the earliest permanent posterior tooth to erupt, seems to be the tooth that most often requires root canal treatment. Anatomical characteristics of permanent mandibular molars are generally described as a group of teeth with two roots. The usual canal distribution is two canals in the mesial root and one or two in the distal root. In 1974, Vertucci and Wiiliams as well as Barker * et al.* described the presence of a middle mesial canal.[3] Since then, several case reports of multiple canal systems in mandibular first molars have been investigated and described.[3–5] It has been postulated that secondary dentin apposition during tooth maturation would form dentinal vertical partitions inside the root canal cavity, thus creating root canals. A third root canal may also be created inside the root canal cavity of mandibular molars by this process. Such third canals are situated centrally between the two main root canals, the buccal and lingual root canals. The diameter of those third middle canals is smaller than that of the other two.[6] The probability of a mandibular first molar having a fifth canal is 1–15%.[5] 

This case report presents the treatment of a mandibular first molar with five root canals, of which three were located in the mesial root. This tooth had three independent canals in the mesial root, a pattern that is seldom encountered.

## CASE REPORT

A 45 year-old female patient presented with a complaint of pain in the posterior right mandibular region for the past two weeks. She gave a history of intermittent pain in the same region for the past three months. Her past medical history was found to be noncontributory. Clinical examination revealed a carious right mandibular first molar (46). The clinical and radiographic findings led to a diagnosis of chronic irreversible pupitis of the right mandibular first molar (46), necessitating endodontic therapy.

**Figure 1:**
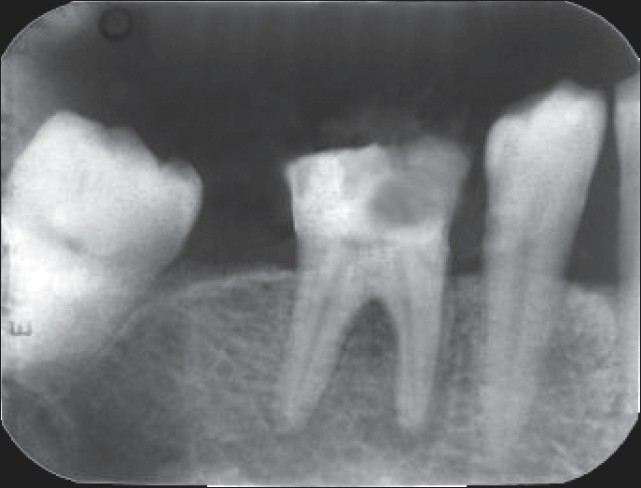
Preoperative radiograph

**Figure 2:**
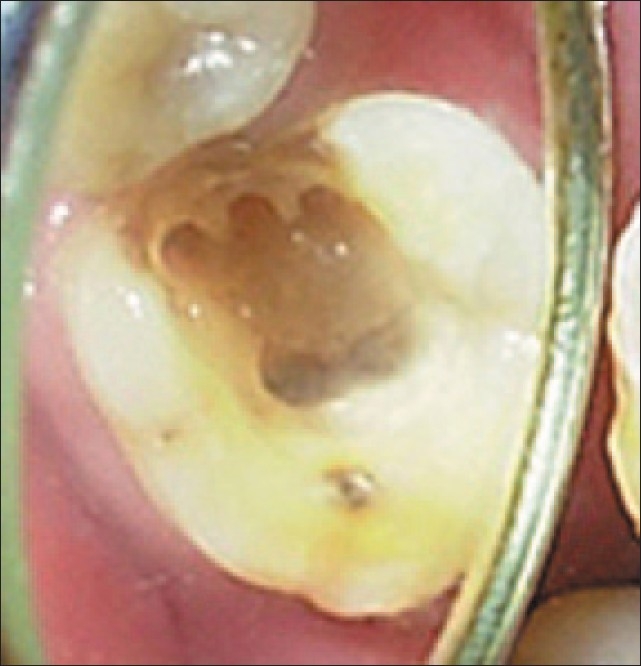
Floor of the pulp chamber showing three mesial and two distal canal orifices

Radiographic evaluation of the involved tooth indicated a normal canal configuration of two canals in the mesial root and one canal in the distal root [Figure 1]. The right inferior alveolar nerve was anesthetized using 2% Lignocaine with 1:80,000 adrenaline (Lignox, Indoco Remedies Ltd, India). The tooth was isolated using a rubber dam and an endodontic access cavity was established. Clinical examination revealed five distinct orifices [Figure 2]: three located mesially (mesiobuccal, middle mesial and mesiolingual) and two distally (distobuccal and distolingual). The canals were explored with a #10 K-file (Mani, Inc; Tochigi, Japan).

**Figure 3:**
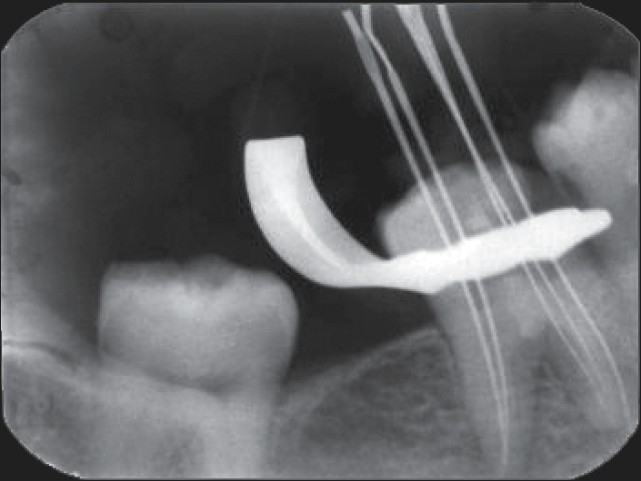
Working length radiograph taken with tube shift (distal shift) technique

**Figure 4:**
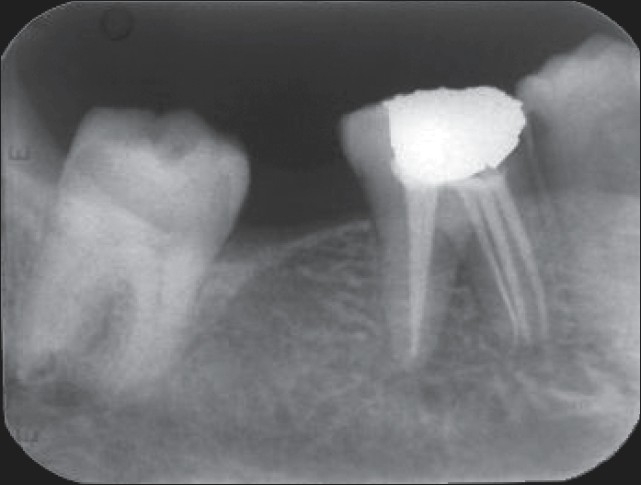
Postoperative obturation radiograph

Multiple, working-length radiographs taken at different angulations with one file placed in each of the three mesial and two distal orifices revealed the presence of five distinct canals [Figure 3]. Cleaning and shaping was performed using a crown down preparation with Protaper series nickel-titanium rotary instruments (Maillefer, Dentsply, Ballaigues, Switzerland) under abundant irrigation with 5.25% sodium hypochlorite solution and EDTA (Glyde, Maillefer, Dentsply, Ballaigues, Switzerland) in a 5 mL syringe. The root canals were dried with paper points (Maillefer, Dentsply, Ballaigues, Switzerland) and obturated with cold, laterally condensed gutta-percha (Maillefer, Dentsply, Ballaigues, Switzerland) and zinc oxide eugenol sealer (Dental products of India Ltd) [Figure 4]. A thin dentine septum separated the two distal canals, which terminated at a common apical foramen, a Vertucci′s type II root canal morphology that overlapped each other on the postoperative radiograph. Three separate apical terminations of the mesial canals with three distinct orifices could be distinguished, a type VIII root canal morphology according to Vertucci′s classification.

## DISCUSSION

A thorough knowledge of root canal morphology and the configuration of the teeth plays an important role in the success of endodontic therapy.[7] Several reports have described the presence of aberrant canals in the mandibular first molar that includes the presence of three canals in the mesial root.[3,4,5,8] The third mesial canal is defined as being independent when a distinct coronal orifice and apical foramen are observed, or as confluent when converging into one of the other two main canals and terminating at a common apical foramen.[4] Table 1 shows the prevalence of a third canal in the mesial root of mandibular first molars. Many authors have agreed on the presence of three foramina in the mesial root, but only a few reported the presence of three independent canals, which presents itself as a rare anatomical variant.[4,9] . In a study of 760 mandibular molars, Fabra * et al* .[10] found that 20 molars (2.6%) had three canals in the mesial root. In 13 (1.7%) of those, the third canal joined the mesiobuccal canal in the apical third of the root and in six (1.6%) molars, the canals converged with the mesiolingual canal, also in the apical third. The third canal ended as an independent canal in only one tooth (0.13%). Goel * et al* .[11] reported that the mesial root of permanent mandibular first molars presented two foramina in 60% of the specimens, whereas 6.7 and 3.3% of these molars had three and four foramina respectively.

Endodontic success in teeth with the aforementioned number of canals requires a careful clinical and radiographic inspection.[12] Diagnostic measures such as multiple, preoperative radiographs, examination of the pulp chamber floor with a sharp explorer, troughing of the grooves with ultrasonic tips, staining the chamber floor with 1% methylene blue dye, performing the sodium hypochlorite “champagne bubble test,” and visualizing canal bleeding points are all important aids in locating root canal orifices.[13] A DG 16 endodontic explorer used as a pathfinder determines the angle at which the canals depart from the main chamber. The search for an extra orifice is also aided by the use of magnifying loupes and fiber-optic transillumination to locate the developmental line between the mesiobuccal and mesiolingual orifices.[14] 

Variations in the mesial root of mandibular first molars can be identified through very careful observation of angled radiographs. Buccolingual views, 20° from mesial and 20° from distal, reveal the basic information on the tooth′s anatomy and the root canal system that is required for endodontic treatment.[15] A significant constraint in conventional radiography is that it produces a 2D image of a 3D object, resulting in the superimposition of the overlying structures. Therefore, these radiographs are of limited value in cases with complex root canal anatomy.[16] Interpretation and appraisal based on a 2D radiograph may alert the clinician to the presence of aberrant anatomy but would not be able to present the variable morphological structure of root canals and their interrelations.[15] Hence, it is mandatory to use all the available diagnostic aids to locate and treat the entire root canal system.[13] Nance * et al*.[17] showed that tuned aperture, computerized tomography imaging enabled a significant increase in canal detection as compared to conventional radiography. Gopikrishna * et al* .[15] used spiral computerized tomography for the confirmatory diagnosis of a morphological aberration in the maxillary first molar.

**Table 1:** Prevalence of a third canal in the mesial root of mandibular first molars according to different authors

Authors	Year	No. of teeth	Method	Thee canals (%)
Skidmore and Bjorndol	1971	45	*Vitco*	0
Pineda and Kuttler	1972	300	*Vitco*	0
Vertucci	1974	100	*Vitco*	1
Pomeranz	1981	100	*Vitco*	12
Martinez-Berna and Badanelli	1983	1418	*Vitco*	1.5
Fabra-Campos	1985	145	*Vitco*	2.1
Fabra-Campos	1989	760	*Vitco*	2.6
Goel	1991	60	*Vitco*	15

Courtesy: Navarro et al.^[5]^

The detection of root canal orifices may be influenced by an anatomical configuration of the root canal system. Also, a better understanding of the root canal anatomy would make it easier to locate canal orifices under magnification, even if the canal anatomy was complicated.[18] The introduction of operating microscope has revolutionized the practice of endodontics. An advantage of using the operating microscope for conventional endodontics is the enhanced visualization of root canal intricacies, which enables the clinician to investigate the root canal system and to clean and shape it more efficiently.[19]

## CONCLUSION

Treating extra canals may be challenging but the inability to find and properly treat root canals may cause failures. Although the incidence of root and canal variations is rare, every effort should be made to find and treat all canals for successful clinical results.
